# The periodontal – endodontic continuum: A review

**DOI:** 10.4103/0972-0707.44046

**Published:** 2008

**Authors:** Raja Sunitha V, Pamela Emmadi, Ambalavanan Namasivayam, Ramakrishnan Thyegarajan, Vijayalakshmi Rajaraman

**Affiliations:** Department of Periodontics, Meenakshiammal Dental College and Hospital, Alapakkam Road, Maduravoyal, Chennai, India

**Keywords:** Classification, combined lesions, perio-endo lesion

## Abstract

Periodontal therapy deals with many aspects of the supporting structures, including the prevention and repair of lesions of the gingival sulcus. Endodontics deals primarily with disease of the pulp and periapical tissues. The success of both periodontal and endodontic therapy depends on the elimination of both disease processes, whether they exist separately or as a combined lesion. The relationship between periodontal and endodontic disease has been a subject of speculation for many years. This paper aims at presenting a comprehensive review of several aspects of perio-endo lesions.

## INTRODUCTION

The tooth, the pulp tissue within it and its supporting structures should be viewed as one biologic unit. The interrelationship of these structures influences each other during health, function and disease. The interrelationship between periodontal and endodontic diseases has aroused much speculation, confusion and controversy. The relationship between the periodontium and the pulp was first discovered by Simring and Goldberg in 1964.[1] The periodontium and pulp have embryonic, anatomic and functional interrelationship. Ectomesenchymal cells proliferate to form the dental papilla and follicle, which are the precursors of the periodontium and the pulp respectively. This embryonic development gives rise to anatomical connections, which remain throughout life.[2]

Three main pathways have been implicated in the development of periodontal-endodontic lesions, namely[3] :

1. Dentinal tubules
2. Lateral and accessory canals
3. Apical foramen

Pulpal and periodontal problems are responsible for more than 50% of tooth mortality.[4] Periodontal disease is a slowly progressing disease that may have an atrophic effect on the dental pulp. Periodontal treatments such as deep root planning, usage of localized medicaments and periodontal injury or wounding may accelerate pulpal inflammation and provoke the interrelated disease process.[5,6]

## PATHWAYS OF COMMUNICATION

Pulpal and periodontal tissues are closely related and the disease transmission between these two lesions has been demonstrated by many studies, which showed significant microbiological similarities between infected root canals and advanced periodontitis.[7–10] Other than these microbial findings, similarities in the composition of cellular infiltrates also suggest the existence of communication between the pulp and the periodontal tissues.[11] These findings infer that cross-contamination between the pulp and periodontal tissues is possible.

**Figure 1:**
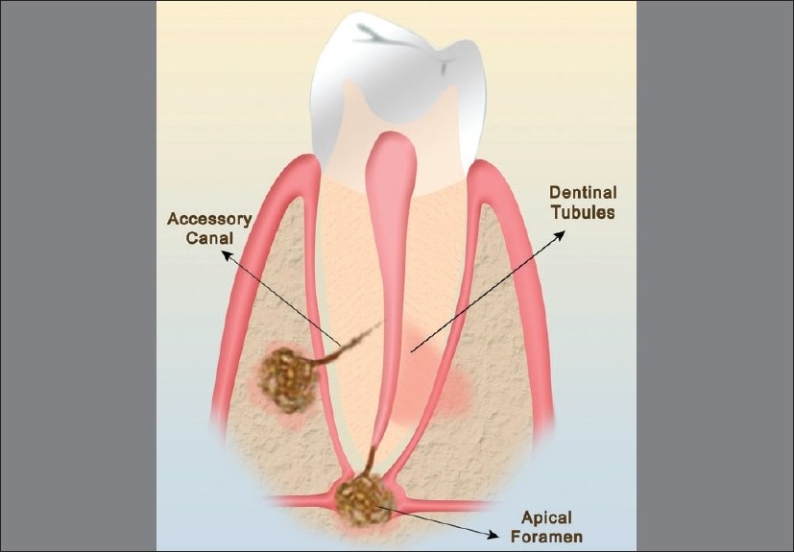
The possible anatomic pathways of communication between the pulp and the periodontium; apical foramen, lateral canals and dentinal tubules

The possible pathways for ingress of bacteria and their products [Figure 1] into these tissues can broadly be divided into: anatomical and nonphysiological pathways.[12]

### Anatomical pathways

The most important among the *anatomical pathways* are vascular pathways such as the apical foramen and the lateral canals and tubular pathways.

*Apical Foramen:* The pulp and periodontal tissues are derived from highly vascular mesenchymal tissues of the tooth germ. The blood supply maintains a connection between these tissues via the apical foramen and lateral canals throughout the development of the tooth. The apical foramen is the principal and most direct route of communication between the periodontium and the pulp. Although periodontal disease has been shown to have a cumulative damaging effect on the pulp tissue, total disintegration of the pulp is only a certainty if bacterial plaque involves the main apical foramen, compromising the vascular supply. Following necrosis of the pulp, various bacterial products like enzymes, metabolites, antigens etc. reach the periodontium through the apical foramen, initiating and perpetuating an inflammatory response there. This results in destruction of periodontal tissue fibers and resorption of the adjacent alveolar bone. External resorption of the cementum can also occur concurrently.

*Lateral canals:* In addition to the apical foramen, which is the main avenue of communication, there are a multitude of branches connecting the main root canal system with the periodontal ligament. These root canal ramifications were first described nearly 100 years ago by Preiswerk (1901). These ramifications are now currently termed as ‘accessory canals’. The term accessory canal is now used to describe any ramification that connects the root canal system to the periodontal ligament.[12] As the root develops, ectomesenchymal channels get incorporated, either due to dentine formation around existing blood vessels or breaks in the continuity of the Hertwigs root sheath, to become lateral or accessory canals.[13] Lateral canals normally harbor connective tissue and vessels which connect the circulating system of the pulp with that of the periodontal ligament. In some instances, the lateral or accessory canal is obliterated by calcification, but patent communications of varying sizes (10-250*µ*m) may remain in many cases. The majority of the accessory canals are found in the apical part of the root and lateral canals in the molar furcation region. The frequency of these ramifications on the root surface are as follows: apical third 17%, coronal third 1.6% and body of the root 8.8%.[14] Bender *et al.*, stated that periodontal endodontic problems were much more frequent in the molars than in the anterior teeth because of the greater number of accessory canals present in the molars. The percentage of lateral canals in the furcation is 46% in first molars[15] and 50 to 60% in any multirooted teeth.[16]

Radiographically, it is seldom possible to identify lateral canals unless they have been filled with a contrasting root canal filling material following endodontic therapy. The radiographic indications of the presence of lateral canals before obturation are:

1. Localized thickening of periodontal ligament on the lateral root surface
2. A frank lateral lesion

It is essential that the dentist recognizes and is familiar with canal ramifications and variations. The ideal treatment of periodontal pocket formation associated with untreated accessory root canals is total debridement and total obturation of the root canal system.[17]

### Tubular pathways

These comprise dentinal tubules which contain the odontoblastic process that extends from the odontoblast at the pulpal dentin border to the dentino-enamel junction or the cement-dentinal junction. Passage of microorganisms between the pulp and periodontal tissues is possible through these tubules, when the dentinal tubules are exposed in areas of denuded cementum.

### Nonphysiological pathways

These include iatrogenic root canal perforations. Improper manipulation of endodontic instruments can also lead to perforation of the root.[12] The second group of artificial pathways between periodontal and pulpal tissues are vertical root fractures, caused by trauma which occurs in both vital and nonvital teeth. The incidence of root fractures is more in the roots that are filled with lateral condensation technique and the teeth restored with intracanal posts.[12]

## ETIOPATHOGENESIS OF PERIO-ENDO LESIONS

### Effect of periodontal lesions on the pulp

**Figure 2:**
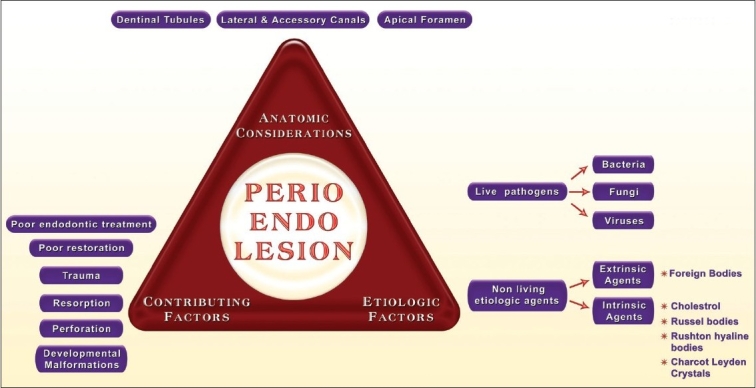
Diagrammatic representation of the etiopathogenesis of endo perio lesions. (Modified from Rotstein I, Simon JHS. Diagnosis, prognosis and decision making in the treatment of combined periodontal-endodontic lesions. Periodontology 2000 2004:34;265-303)

The etiologic factors involved in the evolution of perio-endo lesions can be of a varied nature. However, it is widely accepted that microbial agents are the main cause [Figure 2]. The formation of bacterial plaque on denuded root surfaces, following periodontal disease, has the potential to induce pathologic changes in the pulp through lateral or accessory canals. This process, the reverse of the effects of a necrotic pulp on the periodontal ligament, has been referred to as retrograde pulpitis.[1] The effect of periodontal lesions on the pulp can result in atrophic and other degenerative changes like reduction in the number of pulp cells, dystrophic mineralization, fibrosis, reparative dentin formation, inflammation and resorption.

*Atrophic changes:* The pulp tissue of a periodontally involved tooth has cells which are small and have more collagen depositions than normal. Due to impaired nutrition, the pulp cells slowly degenerate. The death of the cell is so gradual that morphologic evidence sometimes appears to be lacking. The cause of these atrophic changes is the disruption of blood flow through the lateral canals, which leads to localized areas of coagulation necrosis in the pulp. These areas are eventually walled off from the rest of the healthy pulp tissue by collagen and dystrophic mineralization.

With slowly advancing periodontal disease, cementum deposition may act to obliterate lateral canals before pulpal irritation occurs. This may explain why, not all periodontally involved teeth demonstrate pulpal atrophy and canal narrowing. Pressure atrophy may also occur because of mobility of these periodontally involved teeth.

*Inflammatory changes:* The causative agents of periodontal disease are found in the sulcus and are continually challenged by host defenses. An immunologic or inflammatory response is elicited in response to this microbiologic challenge. This results in the formation of granulomatous tissue in the periodontium. When periodontal disease extends from the gingival sulcus towards the apex, the inflammatory products attack the elements of the periodontal ligament and the surrounding alveolar bone.

A clear cut relationship between progressive periodontal disease and pulpal involvement, however, does not invariably exist. The most common periodontal lesion produced by the pulp disease is the localized apical granuloma. It is produced by the diffusion of bacterial products through the root apex, with the formation of vascular granulation tissue. Subsequently, resorption of the alveolar bone and occasionally of the root itself may occur.[17]

*Resorption:* Resorption of the sides of the roots is frequently found adjacent to the granulation tissue overlying the roots. When the periodontal lesions are deep, resorption may also be found within the root canals, often opposite lateral canals, and at the apical foramen. Since this resorptive process extends into the dentin peripherally towards the pulp, and the activating factors are produced from the periodontal lesion, a name which reflects the etiology of this phenomenon, peripheral inflammatory root resorption (PIRR) was proposed.[18]

### Effects of periodontal treatment procedures on the dental pulp

Scaling and root planing – This procedure removes the bacterial deposits. However, improper root planning procedures can also remove cementum and the superficial parts of dentin, thereby exposing the dentinal tubules to the oral environment. Subsequent microbial colonization of the root dentin may result in bacterial invasion of the dentinal tubules.[3] As a consequence, inflammatory lesions may develop in the pulp. The initial symptom is sharp pain of rapid onset that disappears once the stimulus is removed. The increase in intensity of pain may be explained by one or both of the following two reasons. Firstly, the smear layer formed on the root surface by the scaling procedures will be dissolved within a few days. This, in turn, will increase the hydraulic conductance of the involved dentinal tubules[19] and decrease the peripheral resistance to fluid flow across dentin. Thereby, pain sensations are more readily evoked. Secondly, open dentinal tubules serve as pathways for diffusive transport of bacterial elements in the oral cavity to the pulp, which is likely to cause a localized inflammatory pulpal response.[20]

*Acid etching* : Root conditioning using citric acid during periodontal regenerative therapy helps to remove bacterial endotoxin and anaerobic bacteria and to expose collagen bundles to serve as a matrix for new connective tissue attachment to cementum.[11] Though beneficial in the treatment of periodontal disease, citric acid removes the smear layer, an important pulp protector. Cotton and Siegel reported that citric acid, when applied to freshly cut dentine, has a toxic effect on the human dental pulp.[21] However, several other studies have concluded that pulpal changes after the application of citric acid does not show any significant changes in the pulp.[22,23]

### Effects of endodontic infection on the periodontium

It has been demonstrated that intrapulpal infection tends to promote epithelial downgrowth along a denuded dentin surface.[24] Also, experimentally induced periodontal defects around infected teeth were associated with 20% more epithelium than noninfected teeth. Noninfected teeth showed 10% more connective tissue coverage than infected teeth.[25] Therefore, it is essential that pulpal infections be treated first, before undertaking periodontal regenerative procedures.

## CLASSIFICATION OF PERIO-ENDO LESIONS

**Figure 3:**
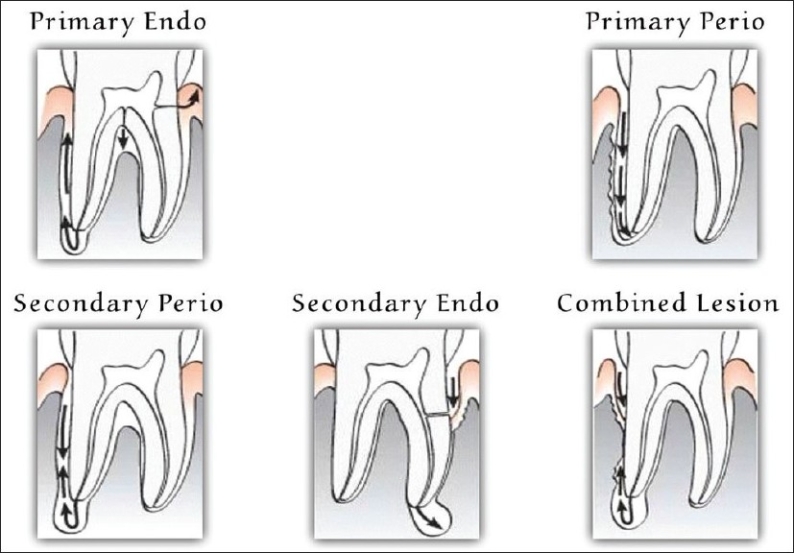
Diagrammatic representation of possible endo perio problems based on the classification of Simon JH, Glick DH, Frank JL 26, 27

A close relationship exists between disease of the dental pulp and periodontal disease, and it expresses itself in several ways. The most commonly used classification was given by Simon, Glick and Frank in 1972 [Figure 3].[26] According to this classification, perio-endo lesions can be classified into:

1. Primary endodontic lesion
2. Primary periodontal lesion
3. Primary endodontic lesion with secondary periodontal involvement
4. Primary periodontal lesion with secondary endodontic involvement
5. True combined lesion

## PRIMARY ENDODONTIC LESION

An acute exacerbation of a chronic apical lesion on a tooth with a necrotic pulp may drain coronally through the periodontal ligament into the gingival sulcus. This condition may clinically mimic the presence of a periodontal abscess. In reality, however, it would be a sinus tract originating from the pulp that opens into the periodontal ligament. Primary endodontic lesions usually heal following root canal therapy. The sinus tract extending into the gingival sulcus or furcation area disappears at an early stage, if the necrotic pulp has been removed and the root canals are well sealed.[3]

## PRIMARY PERIODONTAL LESION

These lesions are caused primarily by periodontal pathogens. In this process, chronic periodontitis progresses apically along the root surface. In most cases, pulpal tests indicate a clinically normal pulpal reaction. There is frequently an accumulation of plaque and calculus and the presence of deep pockets may be detected.[3]

## COMBINED DISEASES

### Primary endodontic lesion with secondary periodontal involvement

If a primary endodontic lesion remains untreated, it may become secondarily involved with periodontal breakdown. Plaque accumulation at the gingival margin of the sinus tract leads to plaque induced periodontitis in this area. When plaque and calculus are detected, the treatment and prognosis of the teeth are different from those of the teeth involved with only endodontic disease. The tooth now requires both endodontic and periodontal treatment.

Primary endodontic lesion with secondary periodontal involvement may also occur as a result of root perforation during root canal treatment, or where pins and posts may have been misplaced during restoration of the crown. Symptoms may be acute, with periodontal abscess formation associated with pain, swelling, pus or exudates, pocket formation, and tooth mobility. A more chronic response may occur without pain, and involves the sudden appearance of a pocket with bleeding on probing or exudation of pus.

Root fractures may also present as primary endodontic lesions with secondary periodontal involvement. These typically occur in root canal treated teeth, often with posts and crowns. The signs may range from a local deepening of periodontal pocket to a more acute periodontal abscess formation.[3]

### Primary periodontal disease with secondary endodontic involvement

The apical progression of a periodontal pocket may continue until the apical tissues are involved. In this case, the pulp may become necrotic as a result of infection entering through lateral canals or the apical foramen. In single-rooted teeth, the prognosis is usually poor. In molar teeth, the prognosis may be better. Since not all the roots may suffer the same loss of supporting tissue, root resection can be considered as a treatment alternative.

If the blood supply circulating through the apex is intact, the pulp has good prospects for survival. It has been reported that pulpal changes resulting from periodontal disease are more likely to occur when the apical foramen is involved. In these cases, bacteria originating from the periodontal pocket are the most likely source of root canal infection.

The treatment of periodontal disease can also lead to secondary endodontic involvement. Lateral canals and dentinal tubules may be opened to the oral environment by scaling and root planning or surgical flap procedures. It is possible for a blood vessel within a lateral canal to be severed by a curette and for the microorganisms to be pushed into the area during treatment, resulting in pulp inflammation and necrosis.[3]

### True combined lesion

True combined endodontic periodontal disease occurs less frequently than other endodontic-periodontal problems. It is formed when an endodontic lesion progressing coronally joins an infected periodontal pocket progressing apically.[26] The degree of attachment loss in this type of lesion is invariably large and the prognosis guarded. This is particularly true in single-rooted teeth. In molar teeth, root resection can be an alternative treatment. The radiographic appearance of combined endodontic periodontal disease may be similar to that of a vertically fractured tooth. If a sinus tract is present, it may be necessary to raise a flap to determine the etiology of the lesion.

## DIAGNOSIS OF PERIODONTAL-ENDODONTIC LESIONS

Diagnosis of primary endodontic disease and primary periodontal disease usually present no clinical difficulty. In primary periodontal disease, the pulp is vital and responsive to testing. In primary endodontic disease, the pulp is infected and nonvital. However, primary endodontic disease with secondary periodontal involvement, primary periodontal disease with secondary endodontic involvement, or true combined diseases are clinically and radiographically very similar. Accurate diagnosis can be achieved by careful history taking, examination and the use of special tests [Table 1]. The extra oral and intra oral tissues are examined for the presence of any abnormality or disease.

**Table 1:** Various diagnostic procedures that can be used to identify perio endo lesions

**Visual examination**
Soft Tissues
Inflammation
Ulcerations
Sinus tracts
Teeth
Caries
Defective restorations
Abrasions
Cracks
Fractures
Discolorations	
**Palpation**
Periradicualr abnormalities
Cannot differentiate between endodontic and periodontal lesion
Compare with control teeth	
**Percussion**
Periradicular inflammation
Compare with control teeth	
**Mobility**
Loss of periodontal support
Fractured roots
Recent trauma
Periradicular abscess	
**Radiographs**
Periradicular bone resorption of endodontic origin - not effective
Bone loss due to periodontal disease - effective	
**Pulp vitality testing**
*(Cold test, Electric test, Blood flow tests, Cavity test)*
Abnormal response – Degenerative changes
No response – Pulp necrosis
Moderate transient response – Normal vital pulp
Quick painful response – Reversible pulpitis
Lingering painful response – Irreversible pulpitis	
**Pocket probing**
Probing depth
Clinical attachment level
Sinus tracking	
**Fistula tracking**
Semi rigid radioopaque material (gutta percha)	
**Cracked tooth testing**
Transillumination
Wedging
Staining

(Modified from Rotstein I, Simon JHS. Diagnosis, prognosis and decision making in the treatment of combined periodontal-endodontic lesions. Periodontology 2000 2004:34;265-303)

The following steps in diagnosis, aids in deciding an appropriate treatment plan:[3]

## VISUAL EXAMINATION

Examination of soft tissues, alveolar mucosa and attached gingiva is done for any inflammation, ulcerations or sinus tracts. The teeth are examined for any caries, defective restorations, erosions, abrasions, cracks, fractures, and discolorations like ‘pink spot’ which is indicative of internal resorption. Magnifying loupes and operating microscope can be used for enhanced magnification and illumination

## RADIOGRAPHS

Radiographic examination aids in detection of carious lesions, extensive or defective restorations, pulp caps, root fractures, periradicular radiolucencies, thickened periodontal ligament and alveolar bone loss. Radiographic changes can be detected only after inflammation or bacterial by-products originating from the dental pulp cause sufficient demineralization of the cortical bone. Initially, periradicular bone resorption from endodontic origin is confined to only cancellous bone. Therefore, it is difficult to identify bone loss caused due to endodontic disease in the initial stages. However, periodontal disease causing alveolar bone loss and the presence of calculus can be effectively detected by radiographs.

## PULP VITALITY TESTING

Teeth with vital pulps will react to cold test with sharp brief pain response that usually does not last more than few seconds. An intense and prolonged pain response often indicates pulpal changes and irreversible pulpitis. Lack of response may indicate pulp necrosis.

## POCKET PROBING

The presence of a deep solitary pocket in the absence of periodontal disease may indicate the presence of a lesion of endodontic origin or a vertical root fracture. Periodontal probing helps in differentiating between endodontic and periodontal disease. It can also be used to track a sinus resulting from an inflammatory periapical lesion that extends cervically through the periodontal ligament space. In periodontal lesions, numerous defects are present throughout the mouth and subgingival calculus can be detected.

## TREATMENT OF PERIODONTAL-ENDODONTIC LESIONS

Before the commencement of any kind of advanced restorative work to treat a perio-endo lesion, the prognosis of the tooth should be considered carefully. Whether there is a functional need for the tooth, whether the tooth is restorable after the lesion has been treated and whether the patient is suitable for a lengthy, costly and invasive treatment are factors that should be taken into consideration. If any of these factors are deemed negative, extraction is the treatment of choice.

When the pulp is nonvital and infected, conventional endodontic therapy alone will resolve the lesion. Surgical endodontic therapy is not necessary, even in the presence of large periradicular radiolucencies and periodontal abscesses. If primary endodontic lesions persist, despite extensive endodontic treatment, the lesion may have secondary periodontal involvement or it may be a true combined lesion.

In case of secondary periodontal involvement, root canal therapy is instituted immediately and the cleaned and shaped root canal is filled with calcium hydroxide paste, which has bactericidal, anti inflammatory and proteolytic property, inhibiting resorption and favoring repair. It also inhibits periodontal contamination of instrumented canals via patent channels connecting the pulp and periodontium before periodontal treatment removes the contaminants. Treatment results should be evaluated after two to three months and only then should periodontal treatment be considered. This allows sufficient time for initial tissue healing and better assessment of the periodontal condition. Prognosis of primary endodontic disease with secondary periodontal involvement depends on periodontal treatment and patient response.[17]

Primary periodontal lesions should be treated first by proper hygiene phase therapy. Poor restorations and any developmental grooves that are difficult to be altered and make oral hygiene maintenance problematic for the patient should be removed. Periodontal surgery is performed after the completion of hygiene phase therapy. Pulpal pathology may be induced while carrying out periodontal therapy in lesions involving the furcation area. Periodontal therapy may consist of procedures that attempt to treat periodontal pockets and promote regeneration. The techniques used include new attachment techniques, gingivectomy, apically displaced flap, removal of the tooth side of pocket by tooth extraction or by hemisection or root resection. In such cases, root canal therapy is not indicated unless pulp vitality test results show change. Re-evaluation must be performed periodically after therapy to check for possible retrograde endodontic problems. The prognosis is entirely dependent on the periodontal therapy, in such cases. Early stage periodontal lesions with secondary endodontic involvement may present as reversible pulpal hypersensitivity, which can be treated purely by periodontal therapy. Periodontal treatment removes noxious stimuli, and secondary mineralization of dentinal tubules allows the resolution of hypersensitivity. If pulpal inflammation is irreversible, root canal treatment is carried out, followed by periodontal treatment. In some cases, periodontal surgical intervention is advantageous.[17]

The prognosis of periodontal lesions is poorer than endodontic lesions and is dependent on the apical extensions of the lesion. A favorable endodontic prognosis is obtained only when the tooth is in a closed and protected environment. As the lesion advances the prognosis approaches that of a true combined lesion.[27]

True combined lesions are treated initially as for primary endodontic lesions with secondary periodontal involvement. Prior to surgery, palliative periodontal therapy should be completed and root canal treatment carried out on the roots to be saved. The prognosis of a true combined perio-endo lesion is often poor or even hopeless, especially when periodontal lesions are chronic, with extensive loss of attachment. Root amputation, hemisection or bicuspidization may allow the root configurations to be changed sufficiently for a part of the root structure to be saved. The prognosis of an affected tooth can also be improved by increasing bony support, which can be achieved by bone grafting and guided tissue regeneration. These advanced treatment options are based on responses to conventional periodontal and endodontic treatment over an extended time period.

Iatrogenic lesions like perforation during root canal instrumentation or preparation of the canal for post and core, require a surgical approach or sealing through an access cavity with a zinc oxide eugenol, glass ionomer or mineral trioxide aggregate sealing material immediately.[28]

## DISCUSSION AND CONCLUSION

The periodontal-endodontic lesion develops by extension of either periodontal destruction apically combining with an existing periapical lesion or an endodontic lesion marginally, combining with an existing periodontal lesion. From the diagnostic point of view, it is important to realize that as long as the pulp remains vital, although inflamed or scarred, it is unlikely to produce irritants that are sufficient to cause pronounced marginal breakdown of the periodontium.

Inflammatory processes in the periodontium associated with necrotic dental pulp and periodontal disease have an infectious etiology. The essential difference between the two disease entities is their respective source of infection. Rarely will established endodontic lesions involve the marginal periodontium, unless they are developing close to the bone margin. A potential pathway for infectious elements in the root canal in such instances may be lateral canals.

Acute manifestations of root canal infections can result in rapid and extensive destruction of the attachment apparatus. Abscesses may drain off in different directions, either through a sinus tract along the periodontal ligament space or through extra osseous fistulation into the gingival sulcus or pocket. Following proper endodontic therapy, these lesions should be expected to heal without a persistent periodontal defect.

Many studies in the literature indicate that combined periodontal and endodontic therapy is essential for successful healing of a periodontal-endodontic lesion. It has been said that either endodontic or periodontic treatment alone would not lead to a satisfactory prognosis, if both disease entities are present and that both must be considered together.[29,30] Hiatt and Amen[31] claimed that persistent periodontal disease may clear up only after definitive periodontal therapy is followed by successful endodontic treatment. Most authors agree that both forms of therapy are essential for successful healing of combined lesions. However, the problem arises over which lesion came first and which caused or perpetuated the clinical problem. It is generally agreed that pulpal disease could initiate or perpetuate periodontal disease; the opposite theory is controversial. Johnson and Orban[32] showed that periodontal disease that remained after unsuccessful endodontic therapy cleared up after successful endodontic therapy. Several authors have also shown the remission of severe periodontal bone loss after endodontic therapy alone. Simring and Goldberg[1] postulated that endodontic therapy is indicated in the treatment of terminal periodontal disease that does not respond to periodontal therapy.

The effect of periodontal inflammation on the pulp is controversial and conflicting studies abound.[11,20,33–36] It has been suggested that periodontal disease has no effect on the pulp, at least until it involves the apex.[34] On the other hand, several studies suggested that the effect of periodontal disease on the pulp is degenerative in nature including an increase in calcifications, fibrosis and collagen resorption, as well as a direct inflammatory effect.[37,38] However, it seems that the pulp is not directly affected by periodontal disease, until recession has opened up an accessory canal to the oral environment. Therefore, treatment of combined lesions should aim at eliminating both the problems.

Treatment and prognosis of primarily endodontic and primarily periodontal disease is very straightforward. However, prognosis of combined forms of the lesions is more difficult to predict. Endodontic therapy is more predictable and completion of this therapy before periodontal procedures has a positive effect on periodontal healing. The most guarded prognosis is given for true combined lesions. In general, assuming that endodontic therapy is adequate, what is of endodontic origin will heal.[3] However, in cases of combined disease, the prognosis of combined diseases rests with the severity and extent of the periodontal lesion and the efficacy of periodontal therapy. In conclusion, it is essential to understand that in perio-endo lesions, the endodontic treatment is the more predictable of the two. However the success of endodontic therapy is dependent on the completion of periodontal therapy. The complete treatment of both aspects of perio-endo lesions is essential for successful long-term results.
